# Dietary and lifestyle indices for hyperinsulinemia with the risk of obesity phenotypes: a prospective cohort study among Iranian adult population

**DOI:** 10.1186/s12889-022-13401-8

**Published:** 2022-05-16

**Authors:** Farshad Teymoori, Ebrahim Mokhtari, Mitra Kazemi Jahromi, Hossein Farhadnejad, Parvin Mirmiran, Mohammadreza Vafa, Fereidoun Azizi

**Affiliations:** 1 Nutrition and Endocrine Research Center, Research Institute for Endocrine Sciences, Shahid Beheshti University of Medical Sciences, Tehran, Iran; 2 Department of Nutrition, School of Public Health, Iran University of Medical Sciences, P.O.Box: 1449614535, Tehran, Iran; 3 Endocrinology and Metabolism Research Center, Hormozgan University of Medical Sciences, Bandar Abbas, Iran; 4 Endocrine Research Center, Research Institute for Endocrine Sciences, Shahid Beheshti University of Medical Sciences, Tehran, Iran

**Keywords:** Obesity phenotype, Hyperinsulinemia, Diet, Lifestyle, Iran

## Abstract

**Background:**

Previous studies have cited insulin-related disorders, including hyperinsulinemia, as one of the main causes of obesity risk and metabolic disorders. We aimed to investigate the association of the Empirical Dietary Index for Hyperinsulinemia (EDIH) and Empirical Lifestyle Index for Hyperinsulinemia (ELIH) with the risk of obesity phenotypes among Iranian adults.

**Methods:**

Present study was conducted on 2705 subjects, including 1604 metabolically healthy normal weights (MHNW) and 1101 metabolically healthy obesity (MHO) individuals. Obesity phenotypes, including MHNW, MHO, metabolically unhealthy normal weights (MUNW), and metabolic unhealthy obesity (MUO), were determined using the criteria of the Joint International statement (JIS) for metabolic syndrome. Dietary intake data from the previous year was gathered using a food frequency questionnaire. Cox proportional hazard regression was used to estimate the hazard ratio and 95% confidence intervals (HRs and 95% CIs) of obesity phenotypes incident across tertiles of EDIH and ELIH scores.

**Results:**

The mean ± SD of age and BMI of all participants were 33.5 ± 12.2 years and 24.3 ± 3.8 kg/m^2^, respectively. In the multivariable-adjusted model, a higher ELIH score was associated with a greater risk for incidence of MUO (HR: 3.47, 95%CI: 2.54–4.74; P_trend_ =  < 0.001) and MHO (HR: 3.61, 95%CI: 2.73–4.77; P_trend_ =  < 0.001). Also, a higher score of EDIH was related to an increased risk of MUO incidence (HR: 1.35, 95%CI: 1.02–1.79; P for trend = 0.046). However, there was no significant association between a higher score of EDIH and the risk of MHO.

**Conclusion:**

Our findings revealed that a high insulinemic potential of diet and lifestyle, determined by EDIH and ELIH indices, may be related to an increase in the simultaneous occurrence of obesity with metabolic disorders in Iranian adults.

**Supplementary Information:**

The online version contains supplementary material available at 10.1186/s12889-022-13401-8.

## Background

Obesity is a chronic condition that affects about 39% of adults globally [1], which reduces life expectancy in individuals with type 2 diabetes (T2D), cardiovascular disease (CVD), and cancer [2]. Evidence suggests a great variation in the risk of metabolic and cardiovascular diseases in obese people that cannot be simply attributed to the amount of body fat mass [3–5]. This evidence also shows that the division of obese and lean individuals based on body weight alone is not correct, and so other factors affecting the risk of cardiometabolic disease should also be considered. These observations led to the concept of four phenotypes based on body mass index (BMI) and the metabolic status (characterized by levels of lipid profiles, blood glucose, blood pressure, and waist), including metabolically healthy obesity (MHO), metabolically unhealthy obesity (MUO) [2], metabolically healthy normal weights (MHNW), and metabolically unhealthy normal weights (MUNW) individuals [6].

Previous studies have cited impaired insulin balance and its-related disorders, including hyperinsulinemia, which may be important causes of increased risk of obesity and another unhealthy metabolic status [7, 8]. Recent reports suggested that specific dietary factors [9, 10] and also dietary patterns or indices that include several dietary factors and cover complex interactions between nutrients and foods may affect insulin homeostasis [11, 12]. Along with diet, body weight and physical activity (PA) are the main other lifestyle factors that are associated with hyperinsulinemia and insulin resistance (IR) [13, 14], therefore, the individual role of diet or its combined role with body weight and PA in the prediction of insulin-related metabolic disorders has been received more attention. In recent years, Tabung et al. have presented two indices to assess the potential of diet and lifestyle to induce or modulate hyperinsulinemia, including Empirical Dietary Index for Hyperinsulinemia (EDIH) and Empirical Lifestyle Index for Hyperinsulinemia (ELIH) [15]. The relationship between these indices and various outcomes has been evaluated so far. Tabung et al. observed that a high EDIH score was associated with long-term weight gain in adults [16]. Also, the positive linkage between these dietary insulinemic indices and risk of IR [17] and T2D [18] has previously been reported.

Although to the best of our knowledge, the association between EDIH and ELIH has not been investigated with obesity phenotypes, some studies have suggested the possible association of other dietary indices with the odds of obesity phenotypes. Farhadnejad et al. showed that a higher DASH score is associated with lower odds of the MUO phenotype [19]. Also, Soltani et al. demonstrated that in obese people, a greater pro-inflammatory diet was linked to a higher risk of unhealthy phenotype. In addition, another study found that the Mediterranean diet is linked with a healthier phenotypic characterization and better metabolic health [20]. Given the growing prevalence of obesity worldwide, preventing obesity-related metabolic disorders is a vital medical matter. So, the present study aimed to investigate the association of dietary and lifestyle indices for hyperinsulinemia with four aforementioned obesity and normal weight phenotypes among Iranian adults.

## Methods

### Study population

The TLGS is a population-based prospective study conducted on 15,005 subjects aged ≥ 3 years among a representative urban population of Tehran. The first examination of the TLGS began in March 1999 to 2001, and data collection, at 3-year intervals, is ongoing.

In the third survey of the TLGS (2006–2008), of 12,523 participants, 3568 were randomly selected for dietary assessment, and in the fourth survey (2009–2011), 7956 randomly selected subjects agreed to complete dietary assessment. In total, 9232 participants, aged ≥ 3 years, with complete dietary data on the third examination of TLGS and the new entries participants in the fourth examination were identified and followed up to the fourth, fifth (2012–2014) and sixth (2015–2018) examination of TLGS.

For the present study, after conducting the exclusion criteria, MHO participants at the baseline (examination 3 or 4) were followed up to the next examinations (examination 4 or 5 or 6). The first metabolic unhealthy (MU) occurrence was identified as the event status. For participants who remained MHO, their last examination was identified as the censored status. Also, those who missed to follow up were excluded from the final analysis.

For this study, of 7773 individuals who have more than 18 years old, at the baseline of study(examination 3 or 4), participants with unhealthy metabolic status (based on metabolic syndrome definition, participants who had two or more metabolic disorders including elevated blood pressure, impaired glucose, hypertriglyceridemia, low high-density lipoprotein cholesterol (HDL-C), and central obesity) (*n* = 4128), prevalent cancer (*n* = 16), cardiovascular accident and myocardial infraction (*n* = 81), pregnant and lactating women (*n* = 196), those with under- or over-reported dietary energy intakes (out of the range 800—4200 kcal/d for men and 500–3500 kcal/d for women) (*n* = 703), and those without data on obesity phenotypes (*n* = 345) were excluded. Some of them may fell into more than one category.

**Fig. 1:**
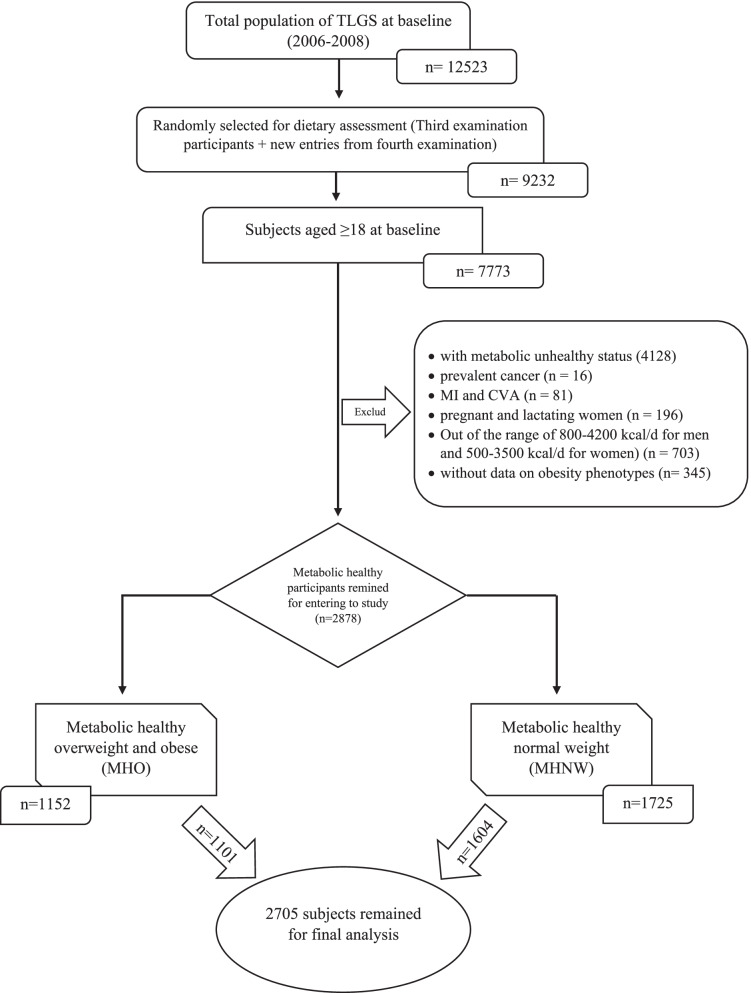
Flow diagram of the study population

Of 2878 healthy metabolic participants at baseline (including 1725 MHNW and MHO subjects), 173 participants were missed to follow up, and for final analysis, 2705 subjects, including 1604 MHNW and 1101 MHO, were followed up by 6.68 years and 5.07 years, respectively. (Follow-up rate: 93.0% for MHNW and 95.5% for MHO participants) (Fig. 1).

### Demographic and anthropometric assessment

Using a pretested questionnaire, an experienced interviewer collected data on age, sex, medical history, medication usage, and smoking habits. Nonsmoker and smoker (ex-smoker, current or occasionally) were the two types of smoking status. As previously described [21], the participant's weight, height, and waist circumference (WC) were measured using a digital scale, stadiometer, and tape meter, respectively. Body mass index was calculated as weight (kg) divided by height in square meters (m^2^).

### Clinical and biological measurements

Details of the study method and measurements have been reported previously [22]. The subject’s systolic blood pressure (SBP) and diastolic blood pressure (DBP) were assessed using conventional methods. After 12–14 h of overnight fasting, a blood sample was taken in a sitting position at the TLGS research laboratory to measure fasting plasma glucose (FPG), triglyceride (TG), and HDL-C.

### Dietary assessment

At the beginning of the study, dietary intake data from the previous year was gathered using a valid and reproducible semi-quantitative food frequency questionnaire [23]. Participants were asked to select their intake frequency for each food item daily, weekly, or monthly during the preceding year; portion sizes of eaten items, given in household measurements, were then translated to grams by trained dieticians.

### Physical activity assessment

We utilized the Modifiable Activity Questionnaire (MAQ), which has previously been updated and validated among Iranians, to gather data on physical activity [24].

### Empirical dietary and lifestyle index for hyperinsulinemia (EDIH and ELIH)

EDIH is calculated based on two groups of food components, including positive (including red and processed meat, margarine, poultry, high-energy beverages, butter, French fries, low-fat dairy, tomatoes, and eggs) and negative (coffee, high-fat dairy, green and leafy vegetables, and whole fruits.) determinants. Each of the mentioned food groups is multiplied by a particular weight that it previously calculated in the study conducted by Tabung and his colleagues [15], and then all food scores were summed as EDIH score.

Similarly, the ELIH is determined based on a set of direct (including BMI, margarine, butter, red meat, and fruit juice) and inverse (including coffee, whole fruits, physical activity, high-fat dairy products, snacks, and salad dressing) components. Like the EDIH, the ELIH score was calculated [15]. The EDIH and ELIH score calculation details were described in a supplementary file.

### Outcome definition

Metabolic health status was defined based on the Joint Interim Phenotypement (JIS) criteria for metabolic syndrome (MetS) [25]. Participants were considered metabolic unhealthy (MU) if they had ≥ 2 of the following: WC ≥ 91 cm in women and ≥ 89 cm in men [26]; FPG ≥ 100 mg/dl or drug treatment; fasting TGs ≥ 150 mg/dl or drug treatment; fasting HDL-C < 50 mg/dl in women and < 40 mg/dl in men or drug treatment; raised blood pressure defined as SBP ≥ 130 mmHg/DBP ≥ 85 mmHg or antihypertensive drug treatment. Those who had one or no component were categorized as metabolic healthy (MH) [19].

We categorized participants based on the BMI < 25 as normal weight (NW) and BMI ≥ 25 as overweight-obese (O). Participants with a BMI ≥ 25 were defined as MHO and MUO if their metabolic status was MH and MU, respectively. Those with a BMI < 25 and defined as MHNW and MUNW if their metabolic status were MH or MU, respectively.

### Statistical analysis

The Statistical Package for Social Sciences (version 20.0; SPSS Inc, Chicago, IL) was used for statistical analyses. Kolmogorov–Smirnov analysis and Histogram charts were conducted to assess the normality of the variables. Baseline characteristics of subjects were expressed as mean ± SD or median (interquartile range, IQR) for continuous variables and percentage for categorical variables, respectively. Independent sample t-test and chi-square analysis were used to assess the statistical differences between normal weight and overweight-obese groups for quantitative and qualitative variables, respectively. Individuals’ duration of follow-up (in year) was calculated from baseline to the time at which an event (definitive diagnosis of a metabolic unhealthy based on the above-mentioned criteria) occurred for the first time (event date) or the last date of follow-up examination, whichever occurred first. The event date of occurrence of a metabolic unhealthy was determined as mid-time between the date of the follow-up visit at which a metabolic unhealthy was detected for the first time and the most recent follow-up visit preceding the diagnosis. Individuals were categorized into tertiles based on the EDIH and ELIH scores.

Cox proportional hazard regression was used to estimate the hazard ratio and 95% confidence intervals (HRs and 95% CIs) of a metabolic unhealthy phenotype incident across tertiles of EDIH and ELIH scores separately in participants with BMI < 25 and BMI ≥ 25. Analyses were adjusted for potential confounders, including age, sex, BMI (only for EDIH), physical activity (only for EDIH), smoking, daily energy intake, and education level. Among MHO participants, we analyzed the risk of MUO across tertiles of EDIH and ELIH. Among MHNW participants, we conducted two separate analyses: we assessed the EDIH and ELIH relation with the incidence of metabolic unhealthy obesity (MUO). Once again, we consider the kind of metabolic unhealthy, including MHO, MUO, and MUNW, as outcomes. We checked the proportional hazards assumption using a log–log plot, and the assumption was satisfied (lines in the plots were parallel). *P*-values < 0.05 were considered as statistically significant.

## Results

During the 6.03 years of follow-up (16,320.4 person-years), 1292 incident cases (47.7%) of the metabolic unhealthy phenotype were identified (the incidence rate = 791.6 per 10.000 person-years) among the total population. Among MHNW participants, the incident cases of metabolic unhealthy including MHO, MUO, and MUNW, were 419, 311, and 322, respectively. The mean ± SD age and BMI of all participants (32.9% male and 67.1% women) were 33.5 ± 12.2 years and 24.3 ± 3.8 kg/m^2^, respectively.

Findings of our study based on the comparison baseline characteristics of participants between the overweight-obese group and normal weight group indicated that the mean age, BMI, WC, FBS, TGs, HDL-C, SBP, DBP, and ELIH score in the overweight-obese group was higher than the normal-weight group (*P*-value < 0.001), however, the individuals in the overweight-obese group have a lower percentage of male, smokers, and EDIH score (*P*-value < 0.001) (Table 1). The results of dietary intakes showed that participants in the overweight-obese group had higher intakes of poultry, high-fat dairy products, whole fruit, green leafy vegetables, and tomato (*P*-value < 0.05) compared to the normal-weight group, however, they had a lower intake of processed meat, French fries, butter, snacks, salad dressing, and high-energy beverages(*P*-value < 0.05) (Table 2).

**Table 1:** Baseline characteristics of 2705 participants of the third and fourth survey of the Tehran Lipid and Glucose Study*

Variables	Total (*n* = 2705)	Normal weight(*n* = 1604)	Overweight-obese (*n* = 1101)	*P*-value
**Demographic-anthropometric data**				
Age (years)	33.5 ± 12.2	30.9 ± 12.1	37.3 ± 11.2	< 0.001
Gender				< 0.001
Men n (%)	890 (32.9)	611 (38.1)	278 (25.1)	
Women n (%)	1815 (67.1)	993 (61.9)	823 (74.9)	
Body mass index (kg/m^2^)	24.3 ± 3.8	21.8 ± 2.1	28.0 ± 2.6	< 0.001
Smoking (%)	258 (9.5)	183 (11.4)	75 (6.8)	< 0.001
Physical activity (MET/hour/week)	60.6 (22.3 – 103.2)	60.6 (23.8 – 104.4)	60.3 (21.0 – 100.9)	0.325
Education level (> 12 years) (%)	721 (26.5)	446 (27.8)	275 (25.0)	0.100
Waist(cm)	80.3 ± 10.4	75.4 ± 8.1	87.5 ± 9.1	< 0.001
**Biochemical-clinical data**				
Fasting blood sugar(mg/dl)	85.6 ± 10.2	84.7 ± 10.0	86.9 ± 10.3	< 0.001
Triglycerides (mg/dl)	91.3 ± 34.4	87.5 ± 34.7	96.8 ± 33.2	< 0.001
HDL- cholesterol (mg/dl)	47.4 ± 10.2	46.6 ± 10.1	48.5 ± 10.1	< 0.001
Systolic blood pressure (mmHg)	104 ± 12	103 ± 12	106 ± 11	< 0.001
Diastolic blood pressure (mmHg)	69.0 ± 8.7	67.7 ± 8.8	70.8 ± 8.3	< 0.001
**Insulin Scores**				
EDIH	0.17 (0.8 – 0.32)	0.18 (0.09 – 0.34)	0.16 (0.07 – 0.28)	< 0.001
ELIH	1.23 ± 0.26	1.10 ± 0.21	1.46 ± 0.22	< 0.001

Data are presented as the mean ± SD or as the median (interquartile range) for continuous variables and as percentages for categorical variables

^*^Significant differences (p < 0.05) were obtained using chi-square for categorical variables and an independent sample t-test for continuous variables

**Table 2:** Dietary intakes of 2705 participants of the third and fourth survey of the Tehran Lipid and Glucose Study*

Variables	Total (*n* = 2705)	Normal weight(*n* = 1604)	Overweight-obese (*n* = 1101)	*P*-value
**Nutrient intake**				
Energy (Kcal/d)	2329 ± 1003	2333 ± 1102	2300 ± 685	0.056
Carbohydrate (% of energy)	57.6 ± 9.4	57.6 ± 10.9	57.5 ± 6.8	0.335
Protein (% of energy)	14.5 ± 7.9	14.4 ± 10.0	14.6 ± 3.1	0.335
Fat (% of energy)	31.6 ± 17.9	31.9 ± 22.6	31.2 ± 6.5	0.302
Saturated fatty acids(% of energy)	10.1 (8.4—12.1)	10.1 (8.4—12.1)	9.9 (8.3—11.9)	0.154
Mono unsaturated fatty acids(% of energy)	10.2 (8.7—12.1)	10.3 (8.7—12.1)	10.2 (8.7—12.1)	0.347
Poly unsaturated fatty acids (% of energy)	5.9 (4.7—7.4)	5.9 (4.8—7.4)	5.8 (4.7—7.2)	0.293
**Food groups**				
Red meat (serving/1000 kcal)	0.05 (0.03 – 0.08)	0.04 (0.02—0.07)	0.04 (0.02—0.07)	0.964
Processed meat(serving/1000 kcal)	0.01 (0.00 – 0.2)	0.01 (0.00—0.02)	0.01 (0.00—0.01)	0.001
Poultry(serving/1000 kcal)	0.06 (0.04 – 0.11)	0.06 (0.03—0.10)	0.06 (0.03—0.11)	0.046
Fish(serving/1000 kcal)	0.03 (0.01 – 0.05)	0.02 (0.01—0.05)	0.02 (0.01—0.05)	0.225
Eggs(serving/1000 kcal)	0.09 (0.05 – 0.16)	0.04 (0.02—0.07)	0.09 (0.05—0.15)	0.864
High-fat dairy products(serving/1000 kcal)	0.61 ± 0.40	0.59 ± 0.39	0.64 ± 0.41	0.001
Low-fat dairy products(serving/1000 kcal)	0.46 ± 0.37	0.45 ± 0.36	0.48 ± 0.38	0.091
Whole fruit(serving/1000 kcal)	0.77 ± 0.55	0.72 ± 0.52	0.84 ± 0.59	< 0.001
Fruit juice(serving/1000 kcal)	0.02 (0.01 – 0.13)	0.01 (0.00—0.05)	0.02 (0.00—0.05)	0.827
Green leafy vegetables(serving/1000 kcal)	0.13 (0.07 – 0.25)	0.11 (0.06—0.23)	0.15 (0.07—0.28)	< 0.001
Tomatoes(serving/1000 kcal)	0.26 (0.13 – 0.43)	0.23 (0.12—0.39)	0.29 (0.16—0.49)	< 0.001
French fries(serving/1000 kcal)	0.00 (0.00 – 0.02)	0.01 (0.00—0.02)	0.00 (0.00—0.01)	0.005
Margarine(serving/1000 kcal)	0.00 (0.00 – 0.00)	0.00 (0.00 – 0.00)	0.00 (0.00 – 0.00)	0.409
Butter(serving/1000 kcal)	0.18 (0.01 – 0.58)	0.22 (0.03—0.66)	0.13 (0.01—0.50)	< 0.001
Coffee(serving/1000 kcal)	0.00 (0.00 – 0.01)	0.00 (0.00 – 0.01)	0.00 (0.00 – 0.01)	0.241
Snacks(serving/1000 kcal)	0.07 (0.01 – 0.20)	0.09 (0.03 – 0.22)	0.06 (0.01 – 0.16)	0.006
Salad dressing(serving/1000 kcal)	0.07 (0.03 – 0.15)	0.08 (0.03 – 0.16)	0.07 (0.02 – 0.14)	0.008
High-energy beverages(serving/1000 kcal)	0.02 (0.00 – 0.06)	0.02 (0.00 – 0.06)	0.01 (0.00 – 0.05)	0.002

Data are presented as the mean ± SD or as the median (interquartile range) for continuous variables and as percentages for categorical variables

^*^Significant differences (p < 0.05) were obtained using an independent sample t-test for continuous variables

We have determined the HRs of MUO across the tertiles of EDIH and ELIH among overweight-obese participants (Table 3). Our findings revealed that there was no significant relationship between the highest vs. lowest tertiles of EDIH and MUO incident in both age and sex-adjusted (HR:1.09, 95% CI:0.90 – 1.30; P for trend = 0.301) and multivariable-adjusted models (HR:1.11, 95% CI:0.92 – 1.34; P for trend = 0.242). However, in the age and sex-adjusted model, the HR of MUO in the highest vs. lowest tertile of ELIH score was 1.30 (95% CI: 1.08–1.56; P for trend = 0.006). After additional adjustment for energy intake, smoking, and education level, the HR of MUO across tertiles of ELIH remained significant (HR: 1.29, 95% CI: 1.08–1.56; P for trend = 0.007).

**Table 3:** The association between insulin indices and incidence of MUO: Tehran Lipid and Glucose Study

	Tertiles of scores			P-trend
	T1	T2	T3	
**EDIH**				
Median score	0.03	0.16	0.37	
Follow up period	5.20 ± 3.32	5.12 ± 3.20	4.91 ± 3.24	
person-years	1909.5	1881.4	1803.4	
Case/Total	239 / 367	226 / 367	239 / 367	
Incidence rate (10.000-person year)	1251 (1102 – 1420)	1201 (1054.3 – 1368)	1325 (1167 – 1504)	
Model 1^a^	1.00 (Ref)	0.97 (0.81 – 1.16)	1.09 (0.90 – 1.30)	0.301
Model 2^b^	1.00 (Ref)	0.99 (0.83 – 1.20)	1.11 (0.92 – 1.34)	0.242
**ELIH**				
Median score	1.19	1.35	1.57	
Follow up period	5.56 ± 3.28	4.92 ± 3.14	4.75 ± 3.29	
person-years	2043.5	1801.9	1748.9	
Case/Total	221 / 367	243 / 366	240 / 368	
Incidence rate (10.000-person year)	1081 (947 – 1233)	1348 (1189 – 1529)	1372 (1209 – 1557)	
Model 1^a^	1.00 (Ref)	1.23 (1.02 – 1.48)	1.30 (1.08 – 1.56)	0.006
Model 2^b^	1.00 (Ref)	1.21 (1.01 – 1.46)	1.29 (1.08 – 1.56)	0.007

^a^Adjusted for age and sex

^b^Additionally, adjusted for body mass index (only for EDIH), physical activity (only for EDIH), smoking, education level, and energy intake

**Fig. 2:**
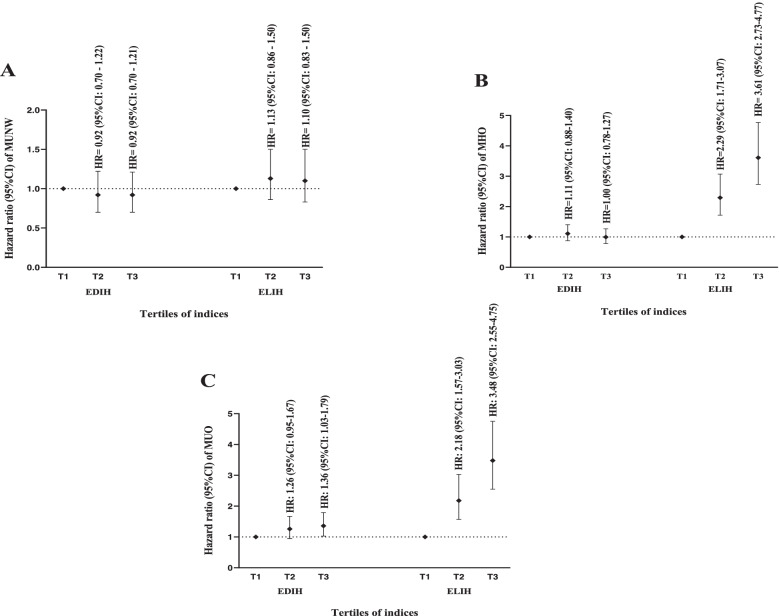
The association between insulin indices and incidence of MUNW, MHO, and MUO among participants of the TLGS

The multivariable-adjusted logistic regression model was used to predict the incidence of MHO, MUO, and MUNW across tertiles of EDIH and ELIH among the metabolic healthy normal weight participants (Fig. 2 A-C). There were no significant association between higher EDIH (HR: 0.91, 95% CI: 0.69 – 1.21; P for trend = 0.587) and ELIH (HR: 1.10, 95% CI: 0.83 – 1.45; P for trend = 0.505) with the risk of MUNW (Fig. 2A). The HRs and 95% CI of MHO for highest vs. lowest tertiles of EDIH and ELIH were 0.99 (0.78 – 1.26), P for trend = 0.875 and 3.61 (2.73 – 4.77), P for trend =  < 0.001, respectively (Fig. 2B). Higher values of EDIH and ELIH were associated with higher risk of MUO incident (HR: 1.35, 95% CI: 1.02 – 1.79; P for trend = 0.046) and (HR: 3.47, 95% CI: 2.54 – 4.74; P for trend =  < 0.001), respectively (Fig. 2C).

We also conducted stratified analyses based on sex group classification (separately among males and females) to assess the association between EDIH and ELIH with different obesity phenotypes and observed no different findings compared to when we analyzed the total population (Data not shown).

## Discussion

In this population-based cohort study, we have assessed the association of insulinemic potential of diet and lifestyle with the risk of obesity phenotypes in the adult population by controlling potential confounding factors. Our findings suggested that a higher score of ELIH was positively related to the risk of MHO and MUO. However, no significant association was observed between ELIH and the risk of MUNW. Also, we showed that higher insulinemic potential of diet, determined by EDIH score, may be associated with increased risk of MUO, but it was not associated with risk of other types of obesity phenotypes, including MHO and MUNW.

Although this study firstly assessed the association of EDIH and ELIH with the risk of obesity phenotypes, our results are comparable with previous investigations conducted on the role of the insulinemic potential of diet and lifestyle with weight gain and metabolic disorders [16, 18]. Tabung et al. revealed that the high insulinemic potential of the diet was associated with a higher risk of long-term weight gain and obesity in adults [16], however, contrary to the results of the above-mentioned study, in our study, no significant association was observed between the higher score of EDIH and MHO risk. In this study, we indicated that adherence to a dietary pattern with higher score of EDIH may increase the risk of obesity with metabolic disorders (MUO phenotypes risk), however, other studies conducted on the Iranian population did not show a strong significant relationship between higher EDIH score and the risk of metabolic disorders, including IR [18] and T2D [17]. Also, our findings on the unhealthy characteristics of the EDIH or ELIH are comparable with the adverse effects of these insulinemic indices in increasing the risk of chronic diseases in other studies. Two studies have reported that the higher insulinemic potential of diet or lifestyle is related to increment risk of various insulin-related malignancies, such as multiple myeloma [27], colorectal cancer [28], and digestive system cancer [29]. Also, Sohouli et al. showed that adult individuals with a higher score of EDIH are more prone to increased risk of non-alcoholic fatty liver disease [30]. An overview of the results of our study and previous evidence suggests that individuals with high insulinemic potential for diet and lifestyle may be more prone to increased risk of chronic diseases such as metabolic syndrome and cardiovascular disease. However, more studies are needed to conclude with greater certainty on the harmful role of these insulinemic indices in increasing the risk of metabolic disorders alongside obesity.

Based on our findings and the results of previous studies conducted on the Iranian population, the EDIH score has shown low potency in predicting the risk of cardiometabolic diseases such as IR, obesity, and T2D. Some reasons can explain this; because of the low consumption of dietary components of EDIH consequently, individuals' estimated scores for this index are low. Also, the amount of individuals' intakes for the food components of EDIH are close to each other and do not have high dispersion, and as a result, the estimated EDIH score in our study population had a narrow range. Moreover, the EDIH index was developed and validated in a different population, which can be one of the causes of this consistency in our results and Tabung et al. study. In our study, a diet with a high EDIH score, although was not associated with the risk of MHO, has been directly linked to an increased risk of MUO; the main reason for this difference in results for the EDIH and obesity phenotypes seems to be that; as reported above, this dietary insulinemic index alone is not a strong indicator for predicting risk of chronic diseases and metabolic disorders in our population, however, in individuals who are genetically predisposed to the risk of metabolic disorders, the combination of this dietary index along with other adverse environmental factors such as elevated body weight may increase the risk of obesity with metabolic disorders in participants with high-risk genetic background.

In the current study, we showed that higher ELIH score, determined by the combination of insulinemic potential of diet, physical activity, and BMI, are strongly associated with increased risk of two abnormal types of phenotypes, including MHO and MUO. Our results are in agreement with the findings of other studies that investigated the association of insulinemic potential of lifestyle with metabolic disorders [18]. Farhadnejad et al. have shown that a higher score of ELIH was related to an increased risk of diabetes by 89% [18]. Also, it has previously been reported that adherence to a lifestyle with a higher score of ELIH may be associated with an increased risk of insulin metabolism-related disorders, including IR, insulin insensitivity, and hyperinsulinemia [18]. Based on the main result of our study, the ELIH score has much higher power than the EDIH score in predicting the occurrence of obesity phenotypes, including MHO (HR: 3.61 vs. HR: 0.99) and MUO (HR: 3.47 vs. HR: 1.35); These are expectable findings because the ELIH was determined by three strong and independent predictors of obesity phenotype including dietary pattern, BMI, and physical activity that all of them have high insulinemic potential individually; indeed, the cooperative contributions of these above-mentioned major lifestyle-related factors to producing the insulinemic effect, suggested a greater association with obesity phenotypes in comparison to the EDIH as an alone insulinemic dietary index. Based on our findings, in participants with normal weight, EDIH and ELIH scores could not predict the increased risk of metabolic disorders without the occurrence of obesity (risk of MUNW phenotype); these results suggest that the insulinemic potential of diet and lifestyle increases the risk of metabolic disorders through weight gain or in combination with it, however, the occurrence of metabolic disorders without increment obesity risk in participants may be due to other factors such as genetic predisposition, high-stress level, and possibly unknown factors.

Our findings suggested that individuals with a higher EDIH score have greater adherence to an unhealthy diet that may contribute to the development of obesity and metabolic disorders. A dietary pattern with a higher score of EDIH is characterized by higher consumption of red and processed meats, margarine, soft drinks, fried potatoes, and refined grains and lower consumption of whole grain, coffee, high-fat dairy, fruits, and leafy green vegetables, which could be strongly effective in insulin secretion, impaired insulin function, and increased its related disorders [31, 32]. Individuals with higher adherence to this dietary pattern are more prone to IR and, consequently, its related metabolic disorders such as MUO [32, 33]. However, a dietary pattern with contrasting features, such as the DASH diet, may have a low potential of insulinemic effect; because this diet mostly focuses on higher intakes of whole grain, plant protein and fats, fiber, calcium, magnesium, and potassium and lower intakes of refined carbohydrates, animal protein and fats, simple sugar, and salt, which can decrease the risk of IR and abnormal types of obesity phenotypes [19, 34].

The elevated BMI as the main determinant of the insulinemic potential of lifestyle is a major effective factor in insulin metabolism and its related problems; because it has been reported that weight gain and adiposity are independently related to the risk of hyperinsulinemia, IR, and metabolic disorders [35], therefore, the remarkable part of the insulinemic effect of ELIH in increasing risk of obesity phenotypes can be caused by high BMI levels of participants. Like the dietary pattern and BMI, physical activity is a strong independent predictor of insulin metabolism-related disorders [36]. Physical inactivity can potentially be related to increment IR, hyperinsulinemia, and its metabolic disorders via increasing inflammatory cytokines, reducing insulin sensitivity, inappropriate effect on body composition and weight gain, and insufficient effects on energy use in various body organs [36, 37]. Therefore, physical inactivity can determine an important part of the high insulinemic effect of an unhealthy lifestyle. Also, the above-mentioned evidence suggested that the collective contributions of the high level of BMI and physical inactivity alongside a high insulinemic potential of diet in the form of ELIH may be a stronger predictor of the risk of insulin disorders and its related metabolic disorders such as obesity phenotypes rather than EDIH.

The results of our prospective study revealed that the insulinemic indices, specially ELIH were, mostly have a significant association with metabolically unhealthy obesity, considering that EDIH and ELIH are primary predictors of hyperinsulinemia in the body, and hyperinsulinemia can also further lead to metabolic disorders; therefore, the possible link of hyperinsulinemia with obesity or its related metabolic disorders may explain the possible role of these insulinemic indices in increasing the risk of some obesity phenotypes. Previously, it was assumed that hyperinsulinemia-induced obesity is caused mostly by an excess calorie intake [38, 39]. However, regardless of calorie intake, Insulin seems to directly affect the spread of fat mass by increasing fat accumulation [40]. Biochemical studies have found that insulin plays a crucial role in regulating lipid accumulation in adipose tissues through inhibition of lipolysis, enhancing fatty acid uptake and lipogenesis, and inducing the expression of genes promoting uptake and storage of lipids [41]. In the long run, insulin signaling can promote adipogenesis by inducing the adipogenic regulators, including C/EBPα and peroxisome proliferator-activated receptor (PPAR) γ [42]. Experimental studies showed that chronic hyperinsulinemia elevates the inflammation in adipose tissue, leading to disruption in pathways involving the lipid metabolism, like de novo lipogenesis in white adipose tissue (WAT) [43], increasing pro-inflammatory cells, including M1 macrophages and NK-cells, and suppressing M2 macrophages, eosinophils and regulatory T-cells, that have anti-inflammatory action. These results were correlated with the altered obesity-associated metabolic phenotype [44]. Mechanistically, an imbalance in M1 and M2 macrophage proportions in WAT promoted iNOS (inducible nitric oxide synthase): arginase-1 imbalance that resulted in extracellular matrix (ECM) deposition and insulin resistance development and consequently obesity [44]. Information supporting the hyperinsulinemia-induced obesity hypothesis is limited in human studies. However, specific racial ethnicities, such as Pima Indians, known to have very high insulin levels have an increased risk of developing obesity [45]. Also, the ability of hyperinsulinemia to predict weight gain and obesity is more indicated in children than adults in different populations [46, 47].

Our study has several strengths. The current study is the first population-based study to investigate the role of the insulinemic potential of diet and lifestyle with the risk of different types of obesity phenotypes in adults with a proper sample size and long follow-up duration. Also, in this study, valid and reliable food-frequency and physical activity questionnaires were used to collect the study population information. However, our study had some limitations. Although similar to other nutritional cohort studies, a valid and reliable FFQ was used to determine the dietary intakes, the possibility of measurement errors is unavoidable. Also, reverse causation is a limitation in the observational study that may be led to bias in the inferences drawn from our study. Considering that the follow-up time of the current study is more than five years, changes in a participant's diet and lifestyle may occur during follow-up, so the baseline diet and lifestyle status individually may not ideally reflect long-term insulin exposure. Furthermore, despite adjusting the confounding effects of various variables in the final statistical analysis, residual confounding effects cannot be excluded due to unknown or unmeasured confounders (such as exogenous hormones and steroids).

## Conclusion

Our results showed that adherence to a lifestyle with a higher ELIH score could be associated with an increased risk of two inappropriate forms of obesity phenotypes, including MHO and MUO. Therefore, these results suggested that a higher ELIH score can be increased the risk of obesity with metabolic disorders in the adult populations. Also, our findings revealed that a diet with a higher score of EDIH may be associated with an increment in MUO risk. However, there was no significant association between the higher score of ELIH and the risk of metabolic disorders in normal-weight adults. Furthermore, a dietary pattern with a higher score of EDIH was not associated with risks of other types of obesity phenotypes, including MHO and MUNW. Further studies are recommended to assess the role of ELIH and EDIH in the risk of obesity phenotypes development and its potential mechanisms.

## Supplementary Information

**Additional file 1. **

## Data Availability

The datasets generated and analyzed during the current study are not publicly available due to the Research Institute for Endocrine Sciences policies, but are available from the corresponding author on reasonable request.
