# Supplementary material for: Dietary and lifestyle indices for hyperinsulinemia with the risk of obesity phenotypes: a prospective cohort study among Iranian adult population
Source: BMC Public Health. 2022 May 16;22:990. doi: 10.1186/s12889-022-13401-8 (PMC9112538; doi:10.1186/s12889-022-13401-8)
Supplement: Supplementary file 1 — Additional file 1. [file 12889_2022_13401_MOESM1_ESM.docx]

The EDIH score was calculated as follows^*^:

EDIH= Red meat * 0.250 + processed meat * 0.199 + margarine * 0.054 + poultry * 0.183 + butter * 0.094 + French fries * 0.581 + other fish * 0.172 + high-energy beverages * 0.104 + tomatoes * 0.095 + low-fat dairy * 0.025 + eggs * 0.124 + coffee * -0.035 + whole fruits * -0.029 + high-fat dairy products * -0.046 + green leafy vegetables * -0.055.

^*^All food groups were included as serving per day.

The ELIH score was calculated as follows^*^:

ELIH: Body mass index (kg/m^2^) * 0.051 + margarine * 0.041 + butter * 0.058 + red meat * 0.089 + fruit juice * 0.042 + coffee * -0.020 + whole fruits * -0.029 + physical activity (MET-h/week) * -0.001 + high-fat dairy products * -0.054 + snacks * -0.024 + salad dressing * -0.059.

^*^All food groups were included as serving per day.
